# Development of High-Density SNP Markers and Their Application in Evaluating Genetic Diversity and Population Structure in *Elaeis guineensis*

**DOI:** 10.3389/fpls.2019.00130

**Published:** 2019-02-12

**Authors:** Wei Xia, Tingting Luo, Wei Zhang, Annaliese S. Mason, Dongyi Huang, Xiaolong Huang, Wenqi Tang, Yajing Dou, Chunyu Zhang, Yong Xiao

**Affiliations:** ^1^Institute of Tropical Agriculture and Forestry, Hainan University, Haikou, China; ^2^Coconut Research Institute, Chinese Academy of Tropical Agriculture Sciences, Haikou, China; ^3^National Research Center of Rapeseed Engineering and Technology and College of Plant Science and Technology, Huazhong Agricultural University, Wuhan, China; ^4^Department of Plant Breeding, IFZ Research Centre for Biosystems, Land Use and Nutrition, Justus Liebig University Giessen, Giessen, Germany

**Keywords:** *Elaeis guineensis*, SNP, population structure, genetic diversity, linkage disequilibrium

## Abstract

High-density single nucleotide polymorphisms (SNPs) are used as highly favored makers to analyze genetic diversity and population structure, to construct high-density genetic maps and provide genotypes for genome-wide association analysis. In order to develop genome-wide SNP markers in oil palm (*Elaeis guineensis*), single locus amplified fragment sequencing (SLAF-seq) technology was performed in a diversity panel of 200 oil palm individuals and 1,261,501 SNPs were identified with minor allele frequency > 0.05 and integrity > 1. Among them, only 17.81% can be mapped within the genic region and the remaining was located into the intergenic region. A positive correlation was detected between the distribution of SNP markers and retrotransposons [transposable elements (TEs)]. Population structure analysis showed that the 200 individuals of oil palm can be divided into five subgroups based on cross-validation errors. However, the subpopulations divided for the 200 oil palm individuals based on the SNP markers were not accurately related to their geographical origins and 80 oil palm individuals from Malaysia showed highest genetic diversity. In addition, the physical distance of linkage disequilibrium (LD) decay in the analyzed oil palm population was 14.516 kb when *r^2^* = 0.1. The LD decay distances for different chromosomes varied from 3.324 (chromosome 15) to 19.983 kb (chromosome 7). Our research provides genome-wide SNPs for future targeted breeding in palm oil.

## Introduction

African oil palm (*Elaeis guineensis*, 2*n* = 32) is a monoecious species of the family palmae (Arecaceae), and may be qualified as “temporally dioecious” because it produces functionally unisexual male and female inflorescences in an alternating cycle on the same plant, resulting in an allogamous mode of reproduction (Adam et al., 2011). It is also an important tropical oil crop grown throughout Southeast Asia, Africa, Central America, and Brazil. Oil palm has the highest oil yield per unit area of all oil crops: more than six times that of peanut, eight times that of soybean, and 10 times that of rapeseed (Huang, 2017). The usage of palm oil can be traced back to 5000 years ago in coastal West Africa, which was supported by the discovery of palm oil in a tomb at Abydos dating back to 3000 BCE by archeologists (Kiple and Ornelas, 2000). This discovery also implies that the Arab traders may bring palm oil from Egypt (Obahiagbon, 2012). The planting history of African oil palm in Malaysia started in the 1870s, when it was introduced in the country as an ornamental tree. In the 1920s, it was cultivated for oil production in wider areas. Currently, African oil palm is commercially cultivated for palm oil production in the tropics, particularly in Indonesia and Malaysia, which produce most of the palm oil in the world. However, there are conflicts between maintaining sustainable palm oil production and coping with the land, energy limitation, and environmental stress. Applying conventional breeding processes to improve yield, quality traits, and stress resistance in oil palm is still slow because of the long life cycle and lacks of genetic homozygosity in current parental breeding materials (Mayes et al., 2000). Developing high-density molecular markers will be useful for identifying molecular markers linked to agronomic traits and breeding and selection processes in oil palm.

Single nucleotide polymorphisms (SNPs) were referred as single nucleotide variations in the genome sequence. SNPs are the most abundant form of DNA variation, and markers developed based on SNPs could reach higher density than any other marker types. At present, the next-generation sequencing (NGS) technology can generate gigantic amount of sequence data, which make the identification of high-throughput SNP makers in a species possible. High-throughput DNA sequencing technology has now been widely applied to develop massive genotyping arrays, which allowed fast and efficient identification of SNP markers for large numbers of individuals (Ganal et al., 2014). Nowadays, SNP markers had a wide application for different purposes in different crops, including identification of plant varieties and cultivars, QTL analysis, construction of high-density genetic map, and genome-wide association analysis (Delourme et al., 2008; Han et al., 2016).

There were different sequencing strategies to genotype a large number of samples (Xia et al., 2009; Lam et al., 2010; Rubin et al., 2010; Xu et al., 2012). Developing SNP markers through deep re-sequencing could provide all variation types on the re-sequenced region and it is expensive. Alternatively, low-depth genome sequencing technology has relatively low cost and research showed that this method is robust for different types of mapping populations derived from organisms with variable quality of genome sequences and is feasible for organisms with large genome sizes and low polymorphisms (Huang et al., 2009). However, this method is dependent on high-quality reference genome sequence and the cost of library construction is high when dealing with large samples. Currently, several methods of reduced-representation genome sequence had been developed, including genotype-by-genotype (GBS; Elshire et al., 2011), restriction-site associated DNA (RAD) sequencing (Bus et al., 2012), reduced representation libraries (RRLs; Lucito et al., 1998; Altshuler et al., 2000; Tassell et al., 2008; Hyten et al., 2010), and single locus amplified fragment sequencing (SLAF-seq). SLAF-seq has been validated and is considered as a fast, accurate, highly efficient, and cost-effective method for developing large-scale SNP and InDel markers (Sun et al., 2013; Zhang et al., 2013). In the study, we took *E. guineensis* as the reference genome and applied SLAF-seq technology for SNPs identification.

Population structure and linkage disequilibrium (LD) analysis are preconditions for further studies of complex agronomic traits in a natural population (Ersoz et al., 2007). Population structure creates genome-wide LD between unlinked loci and arises from different allele frequencies between subgroups in a population, which were caused from factors such as genetic drift, domestication, or background selection (Ersoz et al., 2007). Population structure detected in natural populations can always result in the detection of spurious association, especially in case-control studies (Yu et al., 2006). Many statistic approaches had been used to reduce spurious association between markers and traits, including structure analysis, Q+K mixed model systems, principal components analysis, restricted maximum likelihood, and efficient mixed-model association. LD is the non-random association of allele at different loci in a population and is also prerequisite for association mapping, which determines marker number and density required for a GWAS analysis. LD can be influenced by many factors, including artificial selection, the rate of genetic recombination, mutation rate, genetic drift, the mating system, population structure, and genetic linkage (Xiao et al., 2012). Genetic linkage is a major factor influencing LD. Moreover, LD can be sometimes detected between alleles at different loci without any genetic linkage. Therefore, the pattern of LD in a genome can reflect the population genetic processes which are structuring it (Flint-Garcia et al., 2003).

In this study, we developed genome-wide SNP markers using SLAF-seq technology for an oil palm population with 200 individuals from southern China, Malaysia, Costa Rica, and Africa. The sequence data for each sample were aligned to the *E. guineensis* reference genome (Singh et al., 2013). Genetic diversity, population structure, and LD were estimated by 1,261,501 newly developed genome-wide SNPs. Our research provides a valuable resource for further genome-wide association studies in African oil palm and exploiting rich allelic variation for marker-assisted breeding.

## Materials and Methods

### Plant Materials and DNA Extraction

A total of 200 oil palm individuals were collected from different geographical locations: 21 were collected from southern China (Hainan Province), and these had been introduced from Malaysia to China in the 1960s; 80 were collected from the plateau region of Malaysia; 90 were collected from Costa Rica; and the remaining nine individuals were collected from Africa. Since 2008, the 200 oil palm individuals have been grown as part of the oil palm germplasm resources of the Coconut Research Institute of Chinese Academy of Tropical Agricultural Sciences, Wenchang town, Hainan Province, China. The average temperature and humidity are approximately 23.9°C and 89%. The detailed information for these oil palm individuals is listed in Supplementary Table S1. DNA samples were prepared from young leaves using the mini-CTAB method (Murray and Thompson, 1980). The concentration and quality of the 200 oil palm DNA samples was examined using a Nanodrop 2000 UV-Vis spectrophotometer (NanoDrop, Wilmington, DE, United States). The quantified DNA was diluted to 100 ng/μl for SLAF sequencing.

### Selection of Enzyme Combinations

To obtain more SLAF tags, *in silico* restriction enzyme cutting sites across the oil palm genome were analyzed in order to select restriction enzyme combinations matching the following criteria: (1) the resulting SLAF tags should contain a low percentage of repeat sequences; (2) SLAF tags should be evenly distributed across the genome of *E. guineensis*; (3) simulated fragments must align uniquely to the reference genome; and (4) the number of the resulting SLAF tags ranged from 314 to 414 bp should account for the largest proportion. According to these four criteria, the restriction enzyme combination of *Hae*III + *Hpy*166II was selected. A total of 244,702 SLAF tags were predicted based on *in silico* digestion using the restriction enzyme combination of *Hae*III + *Hpy*166II.

### SLAF Sequencing

Genomic DNA extracted from the spear leaves of each oil palm individual was digested with *Hae*III and *Hpy*166II to obtain SLAF tags. Subsequently, the obtained SLAF tags were used as DNA templates for fragmentation and reparation, dual-index paired-end adapter ligation, PCR amplification, and target fragment selection (Sun et al., 2013). Finally, the processed SLAF tags were sequenced using an Illumina HiseqTM 2500 (Illumina, Inc., San Diego, CA, United States). The information of the barcode or index sequences for the oil palm samples is listed in Supplementary Table S2.

### Evaluation of Data Quality

The raw output produced by the Illumina HiseqTM 2500 was further analyzed for each sample using “Dual-index” software (Kozich et al., 2013). After adapter sequences were removed, raw short reads were assessed by calculating GC contents and Q30 (Q = -10 × log10e; indicating a 0.1% chance of an error). All SLAF reads were clustered based on sequence alignments using BLAT software (Kent, 2002). Polymorphic SLAF tags were determined by comparing sequence variation between different oil palm individuals. SLAF tag sequences were mapped to the whole genome of *E. guineensis* (Singh et al., 2013, the assembled oil palm genome of Version 5) using Burrows-Wheeler alignment (BWA) tool software (Li and Durbin, 2009).

### Identification of SNP Markers

Single nucleotide polymorphism markers were identified for polymorphic SLAF tags using two software programs: GATK (McKenna et al., 2010) and SAMtools (Li and Durbin, 2009). SNP markers consistently identified by both methods were considered to be reliable. Finally, SNPs that match the criteria of a minor allele frequency (MAF) of > 0.05 and integrity > 1 were selected for subsequent analysis.

### Identification of Genes, Putative SSRs, and Retrotransposons

The gene annotation results were downloaded from the National Center for Biotechnology information for the *E. guineensis* genome. The mining of transposable elements (TEs) in the *E. guineensis* genome sequence was done using RepeatMasker (Smit et al., 2013/2015). The SSR analysis software Msatfinder^1^ was used to identify all possible mono-, di-, tri-, tetra-, penta-, and hexa-nucleotide SSRs with a minimum set of 12, 4, 4, 4, 4, and 4 repeats, respectively (Thurston and Field, 2005).

### Population Structure and Linkage Disequilibrium Analysis

Single nucleotide polymorphisms which had low allele frequency (allele frequency < 25% or gene frequency > 75%) and any missing data were eliminated from further analysis. After SNPs pretreatment, a phylogenetic tree was constructed using the MEGA5 software (Tamura et al., 2011). Meanwhile, Bayesian clustering was applied to analyze the population structure of the 200 oil palm individuals using software STRUCTURE (Pritchard et al., 2000). Based on the same set of SNPs, the number of subgroups (K) was predicted from 1 to 10, and the number of ancestors was determined according to the position of the minimum value, with an error rate obtained from cross-validation (CV). Maximum likelihood estimates for the ancestry proportion from each K subgroup for each accession were calculated.

Linkage disequilibrium across the genome of *E. guineensis* was calculated using TASSEL 4.0 software (Bradbury et al., 2007). LD decay for each chromosome was evaluated at a cut-off value of *r*^2^ = 0.1. The *r*^2^ value for a marker distance of 0 kb was assumed to be 1.

### Linkage Disequilibrium Blocks

The LD blocks present in the 200 oil palm individuals were estimated using HAPLOVIEW v4.2 software. The number and size of the LD blocks present on every chromosome were calculated according to established methods (Barrett et al., 2005).

## Results

### SLAF-seq of 200 Oil Palm Individuals

**FIGURE 1:**
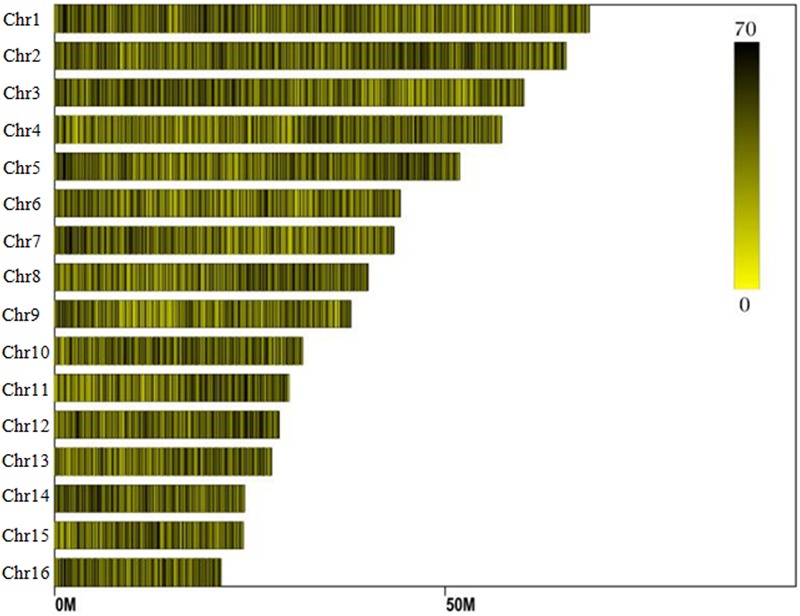
Distribution of SLAF (specific locus amplified fragment) tags across the chromosomes of the oil palm reference genome.

Reads derived from SLAF sequencing of the 200 oil palm individuals were filtered and adaptor sequences removed, resulting in 908.37 Mb of reads with a Q30 of 90.15% and a GC content of 40.79%. A total of 357,378 SLAF tags were obtained from the 200 oil palm individuals, with an average coverage of 10.23 × per sample. SLAF tags were mapped to the reference oil palm genome using the software “BWA,” and 249,457 SLAF tags containing polymorphic SNPs were detected among the 200 oil palm individuals (Table 1 and Figure 1). Of these SLAF tags containing polymorphic SNPs, 43.62% (108,820) were located on the 16 assembled chromosomes of the oil palm reference genome, similar percentage (42.28%) of the total assembled genome sequence of oil palm present in the chromosome assemblies (642.5 Mb of 1.535 Gb). The number of SLAF tags per chromosome ranged from 5 673 (Chr16) to 17 521 (Chr1). A total of 5,870,684 SNPs were identified among the 200 individuals of oil palm, of which 1,261,501 SNPs were identified by both GATK and SAMtools with MAF > 0.05 and integrity > 1 and were subsequently used as SNP markers. The raw data for the 200 oil palm individuals SLAF-seq have been deposited in the European Nucleotide Archive^2^. The bioproject number is PRJEB26466.

**Table 1:** The distribution of SLAF (specific locus amplified fragment sequencing) tags produced across the oil palm (*E. guineensis*) genome.

Chromosome	Chromosome length	SLAF number	Average SLAF distance	The number of polymorphic SLAF
Chr1	68,435,666	17,521	3906	11,048
Chr2	65,558,141	17,080	3838	11,184
Chr3	60,060,032	14979	4010	9785
Chr4	57,251,647	13,691	4182	9055
Chr5	51,955,539	13,228	3928	8574
Chr6	44,357,766	10,302	4306	7252
Chr7	43,454,766	10,697	4062	7218
Chr8	40,195,399	9914	4054	6377
Chr9	38,056,896	8693	4378	5598
Chr10	31,890,735	8380	3806	5627
Chr11	30,068,910	7361	4085	4870
Chr12	28,800,575	7548	3816	5118
Chr13	27,817,470	6989	3980	4650
Chr14	24,379,743	6610	3688	4409
Chr15	24,314,465	6105	3983	4249
Chr16	21,371,083	5673	3767	3806
other	878,010,073	192,607	4559	140,637
**Total**	**1,535,978,909**	**357,378**	**4298**	**249,457**

### Genomic Distribution of the SNPs in *E. guineensis*

Based on the oil palm genome annotation results, 22,957 protein coding genes are distributed on the 16 assembled chromosomes, with gene number per chromosome ranging from 2662 (Chr1) to 812 (Chr16) (date from NCBI). Of the 1.2 million SNP markers located in the 249,457 polymorphic SLAF tags, 17.81% were located within genic regions of 5064 genes (7.38 SNP markers per gene on average). Among these, the largest number of SNP markers (11,319) were located in 2662 genic regions on the chromosome 1, with an average of 4.25 SNP markers per gene, followed by chromosome 2 (10,960 SNP markers in 1769 genes) and chromosome 3 (8491 SNP markers in 1513 genes).

**FIGURE 2:**
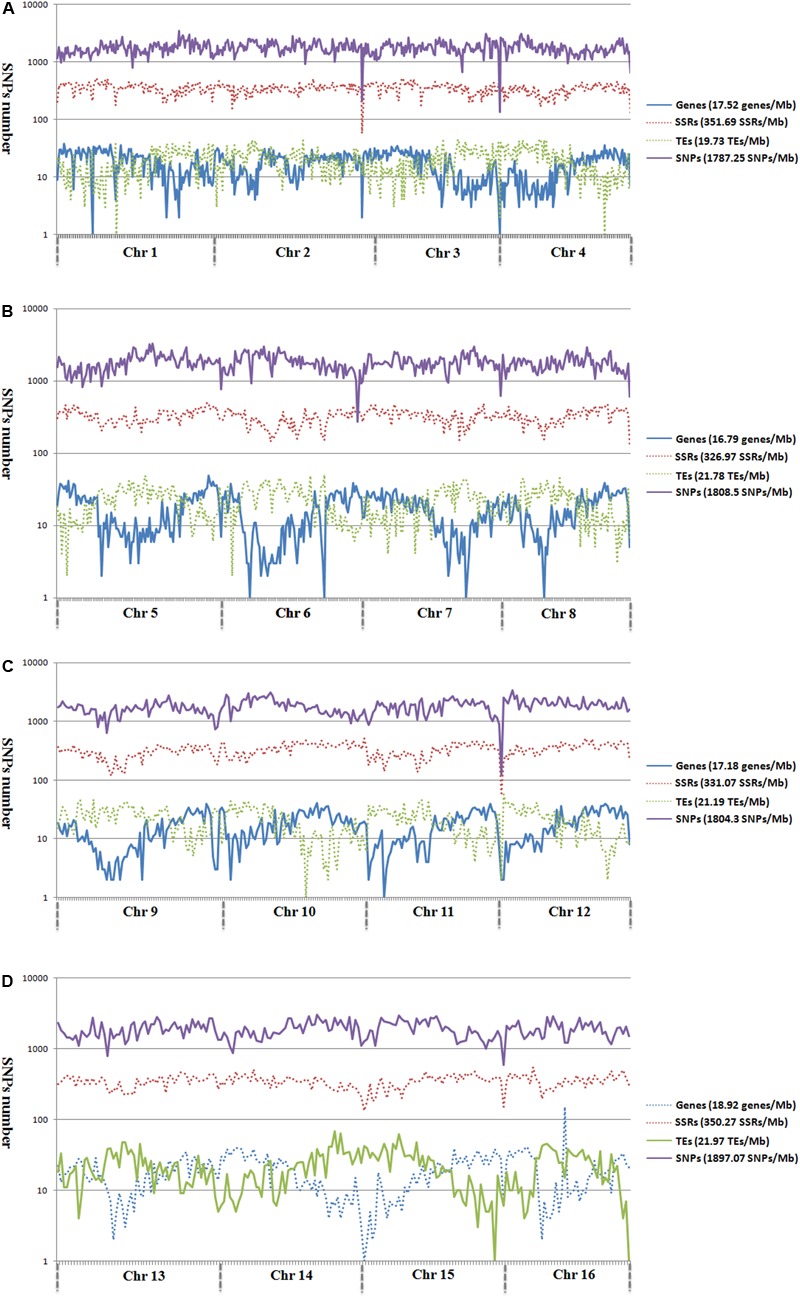
Genomic distributions of genes, microsatellites, TEs, and SNPs across the 16 assembled chromosomes of oil palm, including chromosome 1–4 **(A)**, chromosome 5–8 **(B)**, chromosome 9–12 **(C)**, and chromosome 13–16 **(D)**.

Among the 1,261,501 SNP markers identified in the 200 oil palm individuals, 40.8% were evenly distributed across the 16 chromosomes (Figure 2). The number of SNP markers per chromosome ranged from 17,554 (Chr16) to 52,023 (Chr11), with an average of 32,168 per chromosome.

Analysis of the genomic distributions of SNP markers, microsatellites, TEs, and predicted genes revealed a negative correlation between the distribution of SNP markers and genes (*r* = -0.1355; Figure 2). However, a positive correlation was detected between the distribution of SNP markers and TEs (*r* = 0.3443).

**FIGURE 3:**
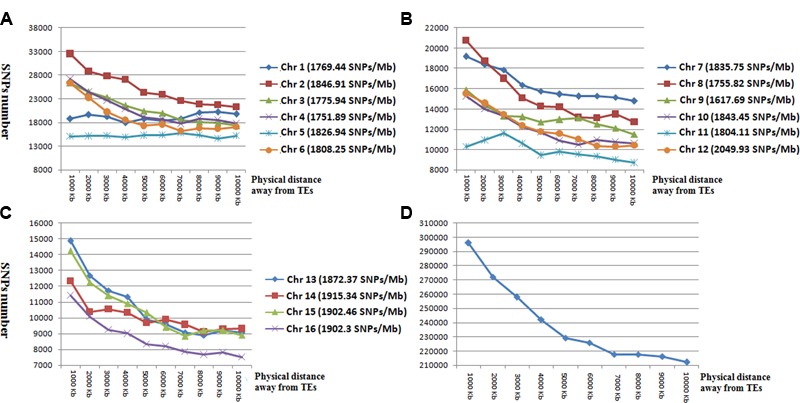
The number and distribution of SNP markers in the 1 to 10-kb flanking region of TEs in chromosomes 1–6 **(A)**, chromosomes 7–12 **(B)**, chromosomes 13–16 **(C)**, and averaged across all chromosomes **(D)**. Horizontal coordinates represent the flanking regions of TEs. Vertical coordinates represent the number of SNP markers.

To determine the relationship between SNP and TEs distributions, we calculated the number of SNP markers in the 1- to 10-kb flanking regions of TEs (Figure 3). SNP distances to TEs were positively correlated with SNP number for all chromosomes except for Chr 1 and Chr 5 (Figure 3). The total number of SNP markers gradually decreased with increasing distance from TEs.

### Population Structure Analysis in Oil Palm

**FIGURE 4:**
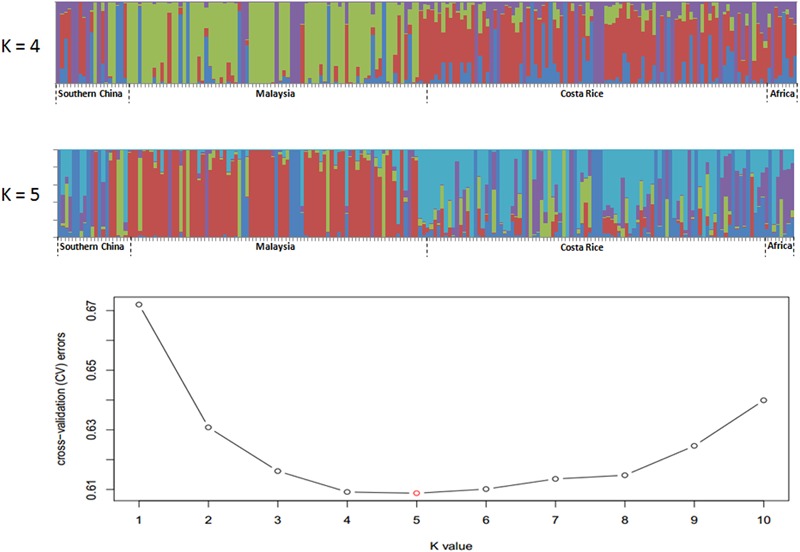
Population structure of 200 oil palm individuals from four different geographical locations. Cross-validation (CV) errors suggest that the 200 oil palm individuals were divided into four or five true genetic populations.

**FIGURE 5:**
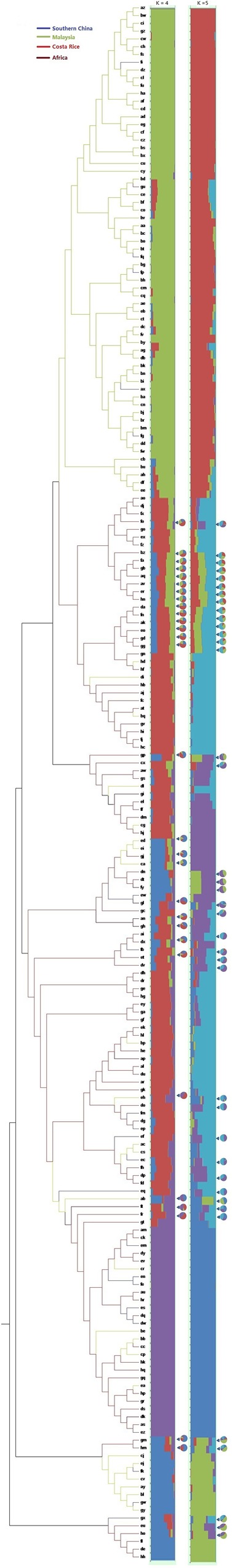
Phylogenetic tree and population structures of 200 oil palm individuals. The maximum likelihood method was applied to assign every oil palm individuals to a cluster, and the cut-off probability was set to 0.6. Colored lines represent geographical origin. Pie chart represents genetic admixture individuals and their genetic composition.

Single nucleotide polymorphisms which had low allele frequency (allele frequency < 25% or gene frequency > 75%) and any missing data were eliminated and a total of 130,414 SNP markers were used for further analysis. We calculated phylogenetic relationship among the 200 oil palm individuals, which is shown in Figure 4. The maximum likelihood method was applied to assign every oil palm individual to a cluster, and the cut-off probability was set to 0.6. Population structure analysis divided the 200 oil palm individuals into four or five subgroups according to CV errors (Figure 4). Comparison of population structures of the 200 oil palm individuals when *K* = 4 or 5 showed that most oil palm individuals from Malaysia were clustered together, along with most oil palm individuals from Costa Rice can basically clustered together. This result was in accordance with the result from the phylogenetic analysis (Figure 5). However, there were genotype admixtures for oil palm individuals from southern China and Africa when *K* = 4 or 5, which was also in accordance with phylogenetic analysis.

The genetic diversity of the 200 oil palm individuals was analyzed (Table 2). The average observed heterozygosity and polymorphism information content was 0.243 and 0.274, respectively. The 80 oil palm individuals from Malaysia had the highest observed allele number (1.996) and observed heterozygosity (0.25), but had a similar level of genetic diversity as the 90 individuals from Costa Rica. Diversity levels were relatively low in individuals from Africa and China.

**Table 2:** Genetic diversity analysis of different populations of 200 oil palm individuals from different geographic origins, including showing observed number of allele numbers, expected number of alleles number, observed number of heterozygous number loci, expected number of heterozygous number loci, and polymorphism information content.

	Southern China		Malaysia		Costa Rica		Africa	
Index	Total	Average	Total	Average	Total	Average	Total	Average
Observed_allele_number	2,470,168	1.96	2,518,412	2	2,518,560	2	2242580	1.79
Expected_allele_number	1,956,294	1.56	1,961,905	1.56	1,973,880	1.56	1874468	1.49
Observed_heterozygous_number	278,424	0.22	322,220	0.26	302,847	0.24	276989	0.22
Expected_heterozygous_number	409,813	0.32	414,824	0.33	421,856	0.33	359851	0.29
Polymorphysm_information_content	329,197	0.26	333,885	0.26	339,491	0.269	286991	0.23

**FIGURE 6:**
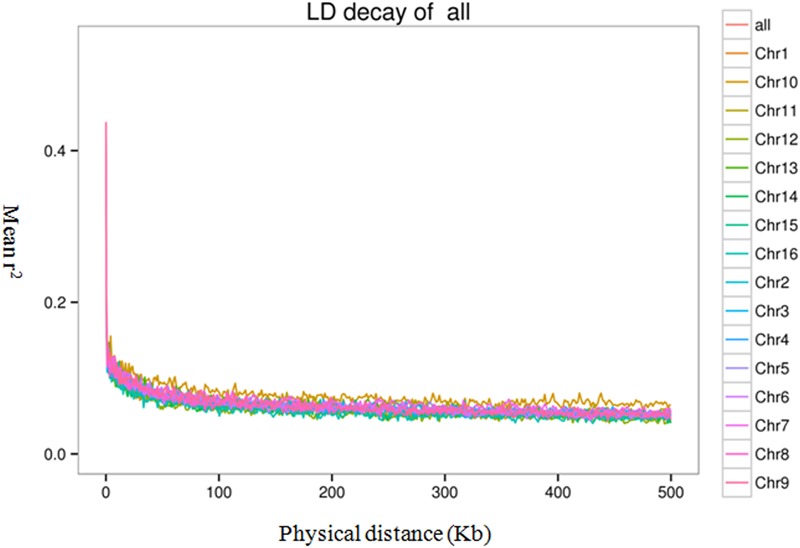
Linkage disequilibrium (LD) decay of the oil palm genome and across different chromosomes.

To evaluate the extent and pattern of LD among the 200 oil palm individuals, we analyzed the LD decay with an *r*^2^ threshold of 0.1. The average physical distance for LD decay was 14.5 kb genome-wide (Figure 6). However, LD decay distances differed among chromosomes and ranged from 3.3 (Chr15) to 20.0 kb (Chr5).

### Haplotype Blocks Based on Linkage Disequilibrium

Haplotype blocks were evaluated based on LD estimation across the 200 oil palm individuals. A total of 9286 conserved haplotype blocks spanning 233.77 Mb (37.11% of the assembled reference genome) were detected among the 200 oil palm individuals. Among haplotype blocks, 54.77% ranged in size from 0 to 1 kb, while 6.98% were >100 kb. The haplotype block number ranged from 280 (Chr16) to 1048 (Chr2), with an average of 580 haplotype blocks per chromosome (Table 3). Haplotype block size per chromosome ranged from 7.79 (chromosome 16) to 23.69 Mb (chromosome 1), with an average of 13.98 Mb (Table 3).

**Table 3:** Distribution of haplotype blocks in the genome of *Elaeis guineensis*.

Chromosome	Chromosome length (Mp)	Number of blocks	Total length of all blocks (Mp)	Frequency
Chr1	68.4	986	23.6	34.60%
Chr2	65.5	1048	23.3	35.60%
Chr3	60.1	872	21.2	35.40%
Chr4	57.2	755	19.2	33.60%
Chr5	51.9	688	18.5	35.70%
Chr6	44.3	577	15	33.90%
Chr7	43.4	655	14.7	34.00%
Chr8	40.1	543	13	32.40%
Chr9	38	433	12.6	33.20%
Chr10	31.8	522	9.9	31.10%
Chr11	30	413	9.4	31.30%
Chr12	28.8	413	9.9	34.50%
Chr13	27.8	402	8.9	32.30%
Chr14	24.3	359	7.3	30.00%
Chr15	24.3	340	8.9	36.60%
Chr16	21.3	280	7.7	36.50%

## Discussion

African oil palm (*E. guineensis*) is an important crop for human nutrition, with tens of millions of tons of palm oil consumed every year. In the study, using SLAF-seq technology, we genotyped a diversity panel of 200 oil palm individuals from four countries and obtained 1.2 million genome-wide SNPs. We then analyzed SNPs distribution in the genome of *E. guineensis* and their relationship with the distribution of TEs, as well as microsatellites and predicted protein-coding genes. Meanwhile, we also used these markers to evaluate the genetic diversity, population structure, and patterns of LD in *E. guineensis*.

Single nucleotide polymorphisms are much more abundant than other molecular markers in the genome, and are considered as highly favored makers for population structure and LD analysis. With the rapid development and application of NGS technology, various reduced-representation genome sequencing approaches have been developed and applied in various species for SNP genotyping. These approaches include GBS (Elshire et al., 2011), RAD sequencing (Bus et al., 2012), RRLs (Tassell et al., 2008), and SLAF-seq, which is an accurate and cost-effective sequence-based SNP identification method (Sun et al., 2013; Zhang et al., 2013). Chen et al. (2013) reported the first use of SLAF-seq and developed 89 specific and stable molecular markers in *Thinopyrum elongaum*. Zhang et al. (2016) reported the construction of a high-density genetic map harboring 5521 SNPs and its application for QTL analysis of boll weight of upland cotton (*Gossypium hirsutum*). Zeng et al. (2017) reported a GWAS study that determined the association between 2,309,777 SNP markers, which were developed via SLAF-seq, and resistance to rust disease in orchard grass. Geng et al. (2016) used SLAF-seq to develop 1933 SNP markers in *Brassica napus*, and subsequently identified four SNP markers that were significantly associated with seed weight. Overall, these results indicated that SLAF-seq is an efficient method for SNP marker identification in a wide variety of crops. In Africa oil palm, reduced-representation genome sequencing approach has been used to develop high-density SNP markers for marker-assisted selections. Pootakham et al. (2015) developed 21,471 SNP markers using GBS method in Africa oil palm and 1085 SNPs were mapped in linkage map. Teh et al. (2015) generated 7.8 million potential SNPs in a population of 132 oil palm individuals by whole genome re-sequencing. In our study, we used SLAF-seq to identify 1,261,501 SNP markers in *E. guineensis*; the average SNP density was 70 SNPs/100 kb, and the average physical distance between adjacent SNP markers was 0.5 kb. In this study, we found that the average LD decay distance was 14.51 kB in our analyzed oil palm population, the large numbers of SNPs obtained via SLAF-seq allowed us to construct a precise haplotype map and conduct a high-resolution association analysis.

We found some correlation between geographic origin and genetic structure among the 200 oil palm individuals. Most oil palm individuals from Malaysia and Costa Rice can each be clustered into a subgroup according to their geographic locations. However, genotype admixtures were detected for oil palm individuals from Southern China and Africa. In addition, genetic diversity was higher among oil palm individuals from Malaysia and Costa Rica than from Africa and China. This was surprising for the African individuals, because oil palm is thought to have originated along the coast of the Gulf of Guinea in Western and Central Africa. On the other hand, the low genetic diversity among African individuals may reflect sampling bias due to the lower number of samples obtained from Africa. The expansion of oil palm as an industrial and food crop began in Africa and Southern Asia (especially Malaysia) during the colonial period. This tropical species was initially introduced into Malaysia as an ornamental tree in the 1870s, but by the 1920s, it was widely cultivated for oil production: Malaysia currently produces 40% of all palm oil worldwide. In the 1960s, the oil palm variety “Dura” was introduced from Malaysia into Southern China (Hainan Province), but initial plantation trials failed because the selected variety was poorly adapted to the climate and soil in Southern China. Subsequent efforts were made to collect oil palm germplasm from different tropical regions and to determine their adaptability to the climate and soil conditions in Southern China, as well as to evaluate their agronomic traits (Cao et al., 2012). However, our results indicate that oil palm in Southern China still has relatively low genetic diversity, suggesting that most oil palm resources are probably derived from the same “Dura” variety introduced from Malaysia in the 1960s. Before the current study, very little was known about the genetic population structure of oil palm, and this has hindered germplasm collection and pre-breeding efforts. Our results increase our understanding of oil palm genetic structure and should therefore enhance pre-breeding and breeding efforts.

African oil palm is native to West and Southwest Africa and originated in the region between Angola and Gambia (Kiple and Ornelas, 2000), Palm oil usage can be dated back to 5000 years ago in coastal west Africa. Currently, the production of palm oil in Southeast Asia has reached 173,390 thousand ton per year, which accounts for more than 80% of world palm oil production (Xie and Zhang, 2009). Since the importance of Africa oil palm, frequency germplasm exchanges have happened between Southern China, African, Malaysia, Costa Rice, and so on.

Africa: Africa oil palm is native to the West and Southwest Africa, especially the area between Angola and Gambia. However, phenotype admixture was detected for oil palm individuals from Africa. Although oil palm originated from Africa, artificial selection has not been used to breed elite oil palm cultivars. Till the 1920s, this tropical crop was introduced into Malaysia for commercial objective. Extensive breed schemes were performed to improve oil yield per unit and oil quality. Hence, recently, elite oil palm cultivar from Malaysia and Costa Rice were introduced back into Africa. The frequent communication between Africa, Malaysia, and Costa Rice is one major reason which causes genotype admixture of oil palm individuals from Africa.

Malaysia: Africa oil palm was initially introduced into Malaysia as an ornamental tree in the 1870s, but by the 1920s, it was cultivated for oil production in Malaysia. Extensive breeding schemes have been used to improve oil yield and quality. Currently, elite oil palm cultivars from Malaysia were introduced into different countries for commercial planting (Lin and Huang, 2002). In our study, oil palm from Malaysia can be classified together.

Costa Rice: Africa oil palm was grown in Costa Rice for commercial objective only 40 years ago. Recently, a large number of elite oil palm germplasm from Malaysia and Africa were introduced into Costa Rice for breeding oil palm cultivar suitable to the plateau region of Costa Rice (Cao et al., 2012). Structure and phylogenetic analysis showed that oil palm individuals from Costa Rice can be basically clustered together.

Southern China: In the 1960s, the oil palm variety “Dura” (thick-shelled) was introduced from Malaysia into Southern China (Hainan Province). Recently, the oil palm variety “Tenera” (thin-shelled) from Malaysia, Costa Rice, and Africa was also introduced into Southern China for adaptation trials (Zhang and Lin, 2006), which may be one major reason causing genotype admixture for Southern China.

## Data Availability

The raw data of 200 oil palm individuals’ SLAF-seq had been deposited onto European Nucleotide Archive (https://www.ebi.ac.uk/ena). The bioproject number is PRJEB26466.

## Author Contributions

YX, WX, and CZ participated in the design of the study. YX and WX had performed the statistical analysis and draft the manuscript. AM critically revised the manuscript. TL did the major experimental work including the extraction of DNA. WZ participated in DNA extraction. WT, YD, DH, and XH contributed to and advised on the statistical analysis. All authors read and approved the final manuscript.

## Conflict of Interest Statement

The authors declare that the research was conducted in the absence of any commercial or financial relationships that could be construed as a potential conflict of interest.

## Footnotes

1 https://github.com/knirirr/Msatfinder

2 https://www.ebi.ac.uk/ena
